# Type 2 myocardial infarction in general medical wards: Clinical features, treatment, and prognosis in comparison with type 1 myocardial infarction

**DOI:** 10.1097/MD.0000000000017404

**Published:** 2019-10-11

**Authors:** Nadav Furie, Ariel Israel, Lee Gilad, Gil Neuman, Fadia Assad, Ilan Ben-Zvi, Chagai Grossman

**Affiliations:** a Department of Internal Medicine F, The Chaim Sheba Medical Center, Tel-Hashomer; b Sackler Faculty of Medicine, Tel-Aviv University; c Department of Family Medicine, Jerusalem Research Center, Clalit Health Services, Jerusalem Region, Israel.

**Keywords:** outcomes, provoking conditions, treatment, type 1 MI, type 2 MI

## Abstract

Type 2 myocardial infarction (MI) is defined as myocardial necrosis due to imbalance between myocardial oxygen supply and demand. The objective of this study was to assess the features, treatments, and outcomes of patients with type 2 MI in comparison with patients with type 1 MI hospitalized in general medical wards. A retrospective review was performed on patients admitted to general medicine wards diagnosed with MI in Sheba Medical Center between January 1, 2016 and December 31, 2016. Comparative analysis between patients with type 1 and type 2 MI was performed. The study included 349 patients with type 1 MI and 206 patients with type 2 MI. The main provoking factors for type 2 MI were sepsis (38.1%), anemia (29.1%), and hypoxia (23.8%). Patients with type 2 MI were older (79.1 ± 11.9 vs 75.2 ± 11.7, *P* < .001) and had a lower rate of prior MI (23.3% vs 38.1%, *P* < .001) and percutaneous coronary intervention (PCI) (34% vs 48.7%, *P* = .023) compared with patients with type 1 MI. Patients with type 2 MI were significantly less prescribed antiplatelet therapy (79.1% vs 96%, *P* < .001) and statins (60.7% vs 80.2%, *P* < .001), and were less referred to coronary angiography (10.7% vs 54.4%, *P* < .001). Type 2 MI was associated with a significantly higher 1-year mortality rate compared with type 1 MI (38.8% vs 26.6%, *P* = .004), but after accounting for age and sex differences, this association lacked statistical significance. In conclusion, type 2 MI patients were older and had similar comorbidities compared with those with type 1 MI. These patients were less prescribed medical therapy and coronary intervention, and had a higher 1-year mortality rate. Establishing a clear therapeutic approach for type 2 MI is required.

## 1 Introduction

Myocardial infarction (MI) is an acute ischemic event associated with cardiomyocyte injury and constitutes a major cause of death and disability.^[1,2]^ The Universal Definition of MI Global Taskforce introduced a classification system in 2007 (and reaffirmed in 2012) that defined 5 types of MI. Type 1 MI is caused by an acute atherothromboembolic coronary event, while type 2 MI is defined as imbalance in oxygen supply and demand which is attributed to a condition other than a coronary atherosclerotic plaque rupture or recent coronary revascularization.^[1]^ In clinical practice, it may be difficult to distinguish type 2 MI from type 1 MI. Hence, large variations in the prevalence of type 2 MI have been reported in the literature, ranging from 1.6% to 29%.^[3–7]^ Type 2 MI is distinguished from myocardial injury without acute ischemia, for example, acute heart failure and myocarditis.^[8,9]^ Type 2 MI is associated with a poor outcome. Several studies have demonstrated higher mortality rates among patients with type 2 MI as compared with patients with type 1 MI.^[3,4,7,10–12]^ Therefore, diagnosis and treatment of patients with type 2 MI is crucial and may have a significant impact on patients’ outcome. Immediate rhythm monitoring, early revascularization, dual-antiplatelet therapy (DAPT), and high-dose statins have all been shown to improve patient outcomes and are therefore uniformly recommended in the current clinical practice guidelines for type 1 MI.^[13]^ Evidence-based treatment recommendations for type 2 MI are lacking and the data regarding the optimal treatment of these patients is limited.^[12]^ Given the complexity of patients with multiple comorbidities hospitalized in general medical wards, type 2 MI is becoming increasingly common. In light of the variance in the clinical features and treatment strategies of patients with type 2 MI, we aimed to depict the clinical features, provoking conditions, treatment and outcome of this sub-group of patients in comparison with patients with type 1 MI hospitalized in general medical wards.

## 2 Methods

Our study is a retrospective cohort study conducted at the Chaim Sheba medical center, a tertiary hospital in Israel. Consecutive patients with acute MI admitted to 7 general medical wards between January 1 and December 31, 2016 were included in the study. Data were retrieved using the electronic medical record database. Admission to general medical departments with a diagnosis of non-ST elevation MI (NSTEMI), ST elevation MI (STEMI), and their synonyms were included. Exclusion criteria were age <18 years and incomplete data. Two reviewers independently inspected each admission and classified it as type 1 or type 2 MI. In the event of disagreement between the 2 reviewers, a third reviewer determined the classification. Data extracted included baseline patient characteristics including demographics and comorbidities, baseline clinical and laboratory features, provoking conditions of type 2 MI, management and outcomes. MI was defined according to the universal definition of MI, with a rise and fall (or a fall alone, when the first measured troponin was also the peak value) of serum troponin above the 99% upper reference limit and clinical evidence of ischemia as defined by at least one of the following: symptoms of ischemia electrocardiogram (ECG) changes with new significant ST-segment–T wave changes, new left bundle branch block (LBBB), or development of pathological Q waves in the ECG; or imaging evidence of a new regional wall motion abnormality.^[1]^ Baseline features included age, sex, body mass index (BMI), and comorbidities. Laboratory data included baseline hemoglobin (Hb) levels, creatinine levels, initial and peak troponin levels. MI characteristics included clinical symptoms (chest pain, dyspnea, syncope, or arrhythmia), baseline blood pressure (BP) and pulse, echocardiography findings, and type of MI (NSTEM/STEMI). Type 2 MI was defined in cases where a comorbid medical condition other than acute coronary artery thrombosis was thought to cause supply/demand mismatch which fulfilled the criteria for acute MI. Provoking conditions for type 2 MI included anemia, sepsis, arrhythmia, valvular disease, congestive heart failure (CHF) exacerbation, non-cardiac surgery and severe hypertension. Multiple provoking conditions were recorded. The following data regarding management of patients were recorded: drug therapy with aspirin, P2Y12 inhibitors, new oral anti-coagulants (NOAC), beta-blockers and statins, performance of cardiac nuclear stress test and percutaneous coronary angiography (PCA), percutaneous coronary intervention (PCI), and coronary artery bypass graft (CABG).

Definitions for conditions provoking type 2 MI were based on a previous classification as follows:

1. Anemia was defined as hemoglobin ≤8 g/dL, gastrointestinal (GI) bleeding, or red blood cell transfusion prior to or within 24 hours following the peak serum troponin.
2. Severe hypertension was defined as a systolic blood pressure above 180 mmHg or a diastolic blood pressure above 110 mmHg.
3. Respiratory failure was defined as the need for high flow oxygen by facemask, non-invasive positive pressure ventilation, or endotracheal intubation and mechanical ventilation.
4. Sepsis was defined as an illness meeting systemic inflammatory response syndrome criteria with an infectious source.
5. Tachycardia and bradycardia were recorded as a provoking condition when the dysrhythmia was suspected as an etiology of myocardial ischemia per the discretion of the treating physician; threshold heart rates for tachycardia or bradycardia were not specified.^[11]^

Outcomes of study population included in-hospital mortality, 30-day mortality, 1-year mortality, and 30-day readmission rates.

The study was conducted according the principles expressed in the declaration of Helsinki and was approved by the institutional review board.

### 2.1 Statistical analysis

Data were analyzed with Statistical R statistical software (The R Foundation for Statistical Computing, Vienna, Austria) version 3.5.1. Continuous variables were expressed as mean ± standard deviation. Categorical variables were expressed as frequencies (percentage). The clinical characteristics, treatments, and outcomes of study subjects were compared with Chi-square tests for categorical variables and independent *t* tests for continuous variables between patients with type 1 and type 2 MI. The probability of death according to MI Type was graphically displayed according to the method of Kaplan–Meier, with comparison of cumulative survival across strata by the log-rank test. Multivariable Cox proportional hazards regression analysis was used to determine the hazard ratio and significance of baseline factors in all-cause mortality. All tests were two-tailed, with *P*-values <.05 being considered as significant.

## 3 Results

MI was diagnosed in 555 patients, 206 (37.1%) of them were classified as type 2 MI. Compared with type 1 MI, patients with type 2 MI were older (79.1 ± 11.9 vs 75.2 ± 11.7, *P* < .001), had a lower rate of prior MI (23.3% vs 38.1%, *P* < .001) prior percutaneous coronary intervention (PCI) (34% vs 48.7%, *P* = .023), and smokers (9.2% vs 17.2%, *P* = .023) (Table 1). Compared with patients with type 1 MI, patients with type 2 MI presented less often with chest pain (42.7% vs 73.9%, *P* < .001), and presented more often with dyspnea, although the difference did not reach statistical significance (59.2% vs 51%, *P* = .07). Patients with type 2 MI had lower baseline Hb levels (10.87 g/dL ± 2.58 vs 12.21 g/dL ± 2, *P* < .001) and higher creatinine levels (1.83 mg/dL ± 1.77 vs 1.52 g/dL ± 1.28, *P* = .01) (Table 2). The main provoking conditions of type 2 MI were anemia (29.1%), sepsis (38.3%), hypoxia (23.8%), and arrhythmia (17%). Forty two percent of the patients had >1 provoking condition (Table 3). Management of patients varied between the groups. Compared with patients with type 1 MI, patients with type 2 MI were less often prescribed anti-aggregate therapy (79.1% vs 96% for aspirin, *P* < .001, 55.3% vs 89.1%, *P* < .001 for P2Y12 inhibitors) and statins (60.7% vs 80.2%, *P* < .001). Compared with patients with type 1 MI, patients with type 2 MI were less often referred to PCA (10.7% vs 54.4%, *P* < .001), and less often underwent PCI (1.5% vs 36.7%, *P* < .001) and CABG (4.6% vs 0%, *P* = .004) (Table 4). In-hospital mortality rates, 30-days mortality rates and 30-days readmission rates were similar between the groups. One year mortality rate was higher among patients with type 2 MI compared with patients with type 1 MI (38.8% vs 26.6%, *P* = .004) (Table 5).

**Table 1:**
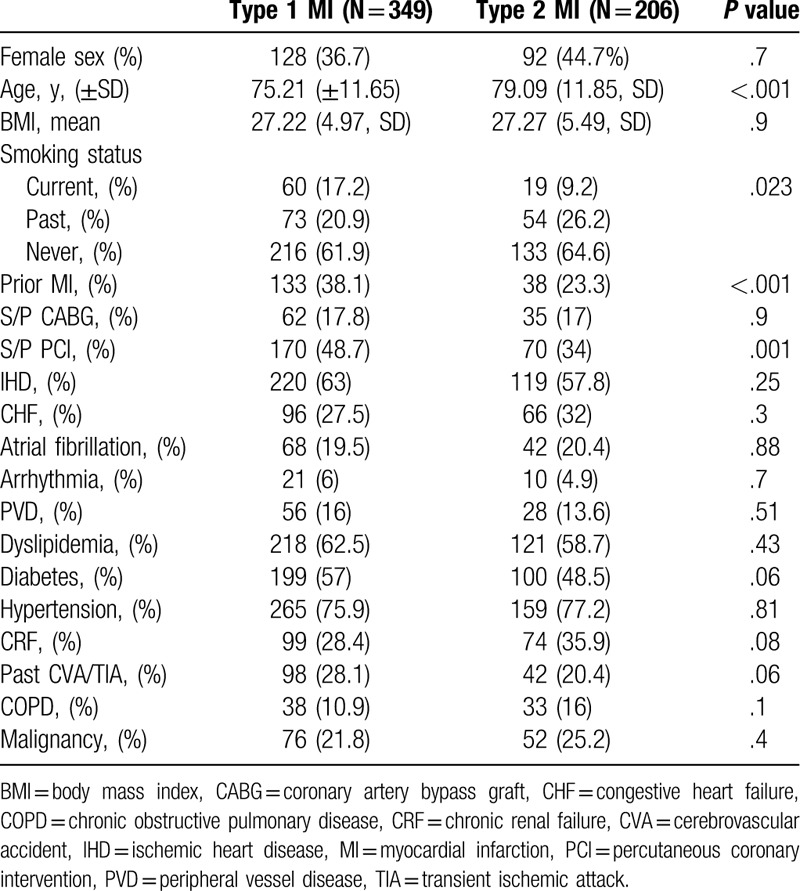
Patient characteristics—comparative analysis between patients with type 1 and type 2 MI.

**Table 2:**
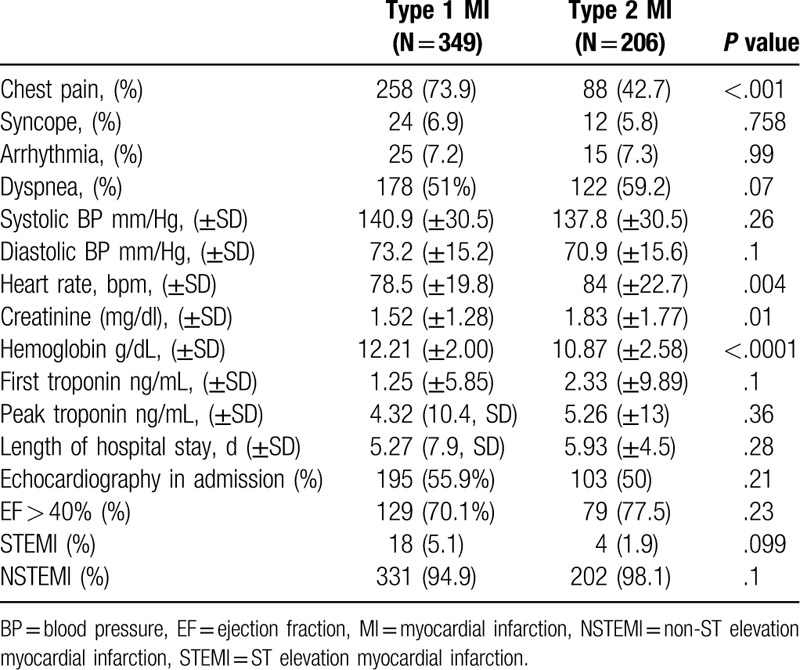
Clinical presentation—comparative analysis between patients with type 1 and type 2 MI.

**Table 3:**
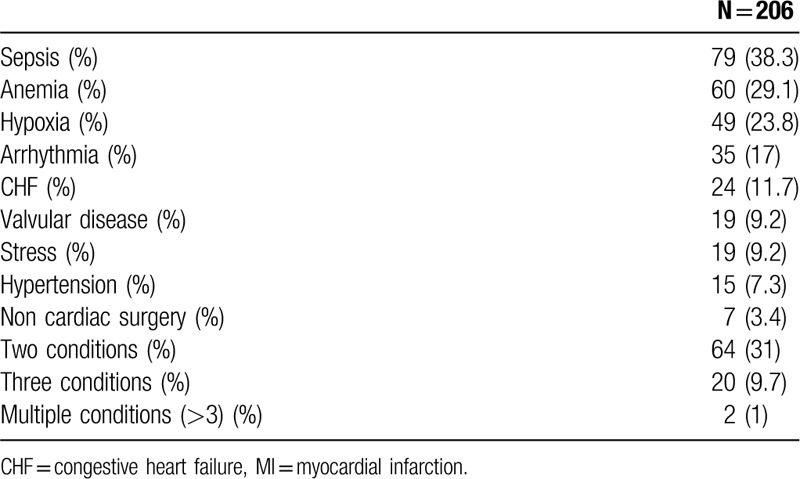
Provoking conditions of type 2 MI.

**Table 4:**
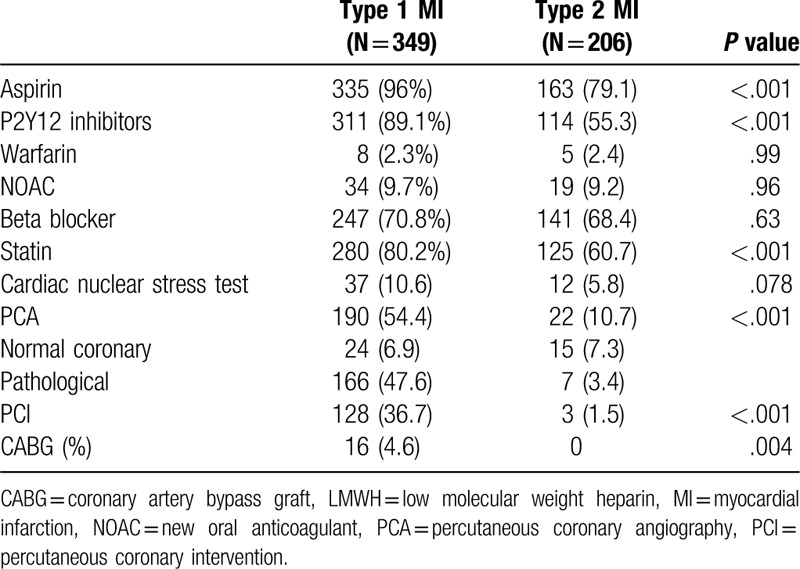
Management of study population—comparative analysis between patients with type 1 and type 2 MI.

**Table 5:**
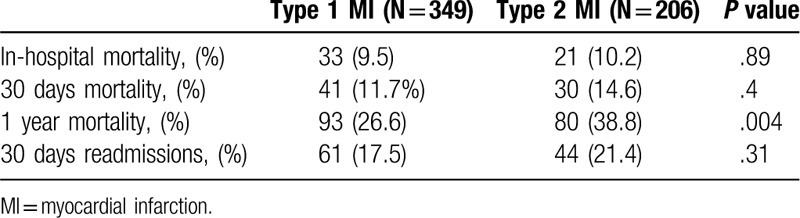
Outcomes of study population—comparative analysis between patients with type 1 and type 2 MI.

**Figure 1:**
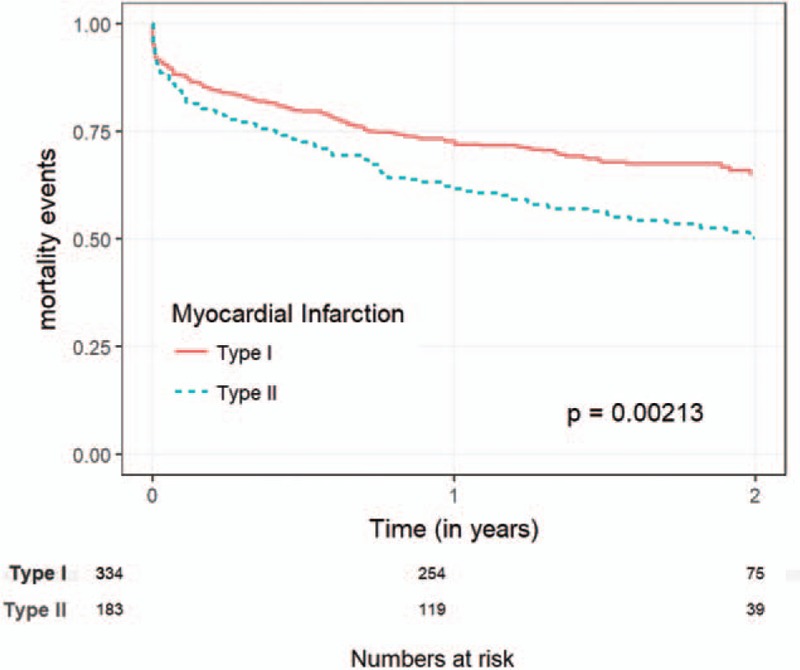
Kaplan–Meier survival analysis, type 1 versus type 2 myocardial infarction.

Kaplan–Meier survival analysis showed overall reduced 1-year survival among patients with type 2 MI (*P* = .002) (Fig. 1). We further performed a Cox regression analysis to detect the main predictors of mortality in our cohort (Table 6). The model showed that increased age, heart rate, and peak troponin value, as well as presence of CHF were associated with increased mortality. After correcting for these factors, MI type was not associated with a significant change in mortality rate.

**Table 6:**
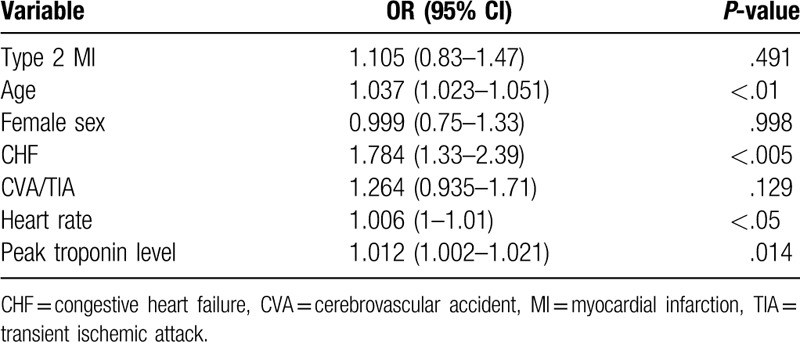
Baseline predictors for 1-year mortality—multivariate regression analysis.

## 4 Discussion

In the present study, 37.1% of patients with MI were classified as type 2 MI. The rate of type 2 MI among all patients with MI ranged from 1.6% to 24.4% in previous cohorts.^[4,5,7,12,14]^ The wide range indicates lack of valid criteria for diagnosis of type 2 MI. In clinical practice, diagnosis of type 2 MI is often determined by the clinical judgment of the treating physician. Additionally, distinguishing type 2 MI from myocardial necrosis not meeting the definition of MI is often difficult. In our study, the proportion of patients classified as type 2 MI was relatively high. This may be attributed to the fact that our study population consisted solely of patients hospitalized in general medical wards, as opposed to previous studies which included unselected hospital cohorts or patients hospitalized in cardiology wards.^[4,5,7,12,14]^ Patients presenting in the emergency room thought to have type 2 MI are usually hospitalized in general medical wards, in order to treat the presumed precipitating factor, for example, sepsis, anemia, arrhythmia, etc. In addition, type 2 MI often develops in the context of medical conditions of patients hospitalized in general medical wards. This may explain the high rate of type 2 MI in our study as compared with previous studies. Noteworthy that a significant number of patients with type 1 MI were hospitalized in general medical wards in our study. This is mainly due to logistic reasons, since the capacity of cardiology wards is limited. Another point worth noting is the fact that several patients with STEMI were hospitalized in general medical wards. Obviously, the vast majority of patients with STEMI undergo primary PCI and are admitted to cardiology units. However, patients who are not candidates for PCI, mainly because of their baseline medical background and cognitive state, are hospitalized in general medical wards and treated conservatively. Indeed, all the patients who underwent a STEMI in our study were managed conservatively and did not undergo PCI.

We observed several differences between the baseline patient characteristics of patients with type 1 and type 2 MI. Patients with type 2 MI were older than patients with type 1 MI. In addition, patients with type 2 MI had lower rates of prior MI and PCI in compared with patients with type 1 MI. These findings are partially compatible with findings in previous studies. Most previous studies have found that patients with type 2 MI were older and had more comorbidities compared with patients with type 1 MI.^[4,7,12]^ Landes et al^[14]^ found similar baseline characteristics including age between type 1 and type 2 MI patients. However, they did observe a higher rate of ischemic heart disease among patients with type 2 MI. Saaby et al^[7]^ found a similar rate of prior MI and PCI among patients with type 1 and type 2 MI. These findings are inconsistent with the findings in our study. The inconsistency between the studies may be attributed to the different inclusion criteria. Our study consisted solely of patients hospitalized in general medical wards. In a relatively high rate of the patients, MI was not the primary diagnosis, but rather secondary to another medical condition. This may explain the finding that the rates of traditional cardiovascular (CV) risk factors and history of coronary heart disease (CHD) were not higher in type 2 MI patients compared to type 1 MI patients in our study.

A previous meta-analysis of observational studies by Gupta et al. comparing type 1 and type 2 MI found that type 2 MI was more common in females.^[15]^ This finding was not observed in our study. Although the rate of females was higher among patients with type 2 MI compared to that of type 1 MI, the difference was not statistically significant. The inconsistency between the studies may be due to the relatively small sample size in our study in comparison with that of Gupta's study.

Previous studies have shown that type 1 MI patients have higher initial and peak troponin levels.^[7,10]^ In our study, initial and peak troponin levels were comparable between type 1 and type 2 MI patients. This difference may be attributed to the fact that our study consisted of patients hospitalized in general medical wards. It is possible that patients with type I MI with a more severe clinical presentation are hospitalized in cardiology units. It may be assumed that these patients have significantly higher troponin levels than those hospitalized in general medical wards.

In our study, clinical presentation was different between patients with type 1 and type 2. Patients with type 1 MI mostly presented typically with chest pain, whereas patients with type 2 MI presented atypically, mainly with dyspnea. This finding is in line with previous studies,^[4,7,12,14]^ and underscore the need to be alert for atypical findings indicating myocardial infarction among patients with complex medical conditions hospitalized in general medical wards. In our study hemoglobin levels were lower and pulse rate was higher among with type 2 MI patients compared to type 1 MI patients. These findings are somewhat expected, since type 2 MI was precipitated by sepsis and anemia in a large proportion of patients in our study.

The main provoking conditions for type 2 MI in our study were sepsis, anemia and hypoxia. Nearly forty percent of the patients had more than one provoking condition. These findings are partially compatible with previous studies. Stein et al^[4]^ have also found anemia and sepsis to be the leading causes of type 2 MI. However, the rate of patients with more than one cause was approximately 20%, significantly less than the rate in our study. Saaby et al^[7]^ observed >1 triggering condition in 11% of the patients with type 2 MI. Baron's group found tachyarrhythmia, heart failure, and sepsis as the most common discharge diagnoses in patients with type 2 MI, assuming that they most likely represent the triggering mechanism.^[12]^

The relatively high rate of multiple triggers for type 2 MI in our study may reflect complex medical conditions of patients hospitalized in general wards. In clinical practice, it is often difficult to determine with certainty the main provoking factor for type 2 MI.

Therapeutic strategies differed between patients with type I and type 2 MI in our study. Patients with type 2 MI were lest often prescribed aspirin, P2Y2 inhibitors and statins. The lower rate of anti-aggregate therapy may be partially attributed to the fact that anemia was a provoking factor in 29% of the patients with Type 2 MI. Prescribing anti-aggregate therapy in these cases, particularly when the cause of the anemia is iron-deficiency, is not indicated and may be harmful. The significantly lower rate of statin therapy is consistent with previous studies.^[7,12]^ Landes et al^[14]^ have previously demonstrated that angiography unmasked acute plaque rupture in 29% of type 2 MI patients. Since classification of MI as type 2 is based on the discretion of the treating physician, it is possible that patients classified as type 2 MI actually have type 1 MI. Given that, it seems that these patients are under-treated with statins, which have a major role in plaque stabilization and reduction of further CV events.^[16–18]^ It seems prescribing statin therapy for patients with type 2 MI should be considered, at least until further cardiac evaluation is performed.

Coronary angiography was performed in 10.7% of patients with type 2 MI in our study, a rate lower than rates reported in previous studies, which ranged between 25% and 36%.^[4,7,11,12]^ Approximately 70% of the patients referred to coronary angiography were found to have normal coronary arteries, and only 13% of them underwent PCI. The variation in the therapeutic approach of patients with type 2 MI stems from the lack of established recommendation regarding this clinical condition. At present, most of type 2 MI patients are managed conservatively, based on the assumption that the triggering factors should be treated and stabilized. Risk stratification of type 2 patients according to triggering factors should be considered, in order to select patients who would benefit from an invasive strategy.^[19–21]^ In our study, in-hospital mortality, 30-days mortality, and 30-day readmission rates were similar between type 1 and type 2 MI patients. However, 1-year mortality rate was higher among type 2 MI patients. Stein et al^[4]^ observed higher 30-day and 1-year mortality rates among type 2 MI patients compared with type 1 MI patients. Saaby et al^[7]^ found type 2 MI as a risk factor for mortality, independent of the triggering conditions leading to the MI. In contrast, Baron et al^[12]^ found that the adjusted mortality rates were similar between type 1 and type 2 MI patients. This latter finding is compatible with the findings in our study, since after accounting for age and sex differences, no association was found between the type of MI and mortality.

A previous meta-analysis of observational studies by Gupta et al^[15]^ comparing type 1 and type 2 MI found that type 2 MI is associated with worse short and long-term outcomes. However, some of the studies included in the meta-analysis did not adjust mortality rates for age and comorbidities. Therefore, the association between type of MI, independent of age and comorbidities, cannot be determined with certainty.

Studies assessing the association between specific therapeutic strategies and outcome of type 2 MI patients are indicated.

Our study has several limitations. First, definition of type 2 MI was based on the clinical judgment of the treating physician. It is possible that misclassification occurred. Secondly, we collected information regarding treatments during hospitalization and recommendations at discharge. We did not have information regarding outpatient treatments following hospitalization. Third, our study consisted solely of patients hospitalized in general medical wards. It is possible that different treatment strategies and outcomes would be observed among patients hospitalized in cardiology and intensive care units. The aim of the study was to focus on the features of type 2 MI patients hospitalized in general medical wards.

Type 2 MI complicating complex medical conditions among patients with multiple comorbidities hospitalized in general medical wards constitutes a major diagnostic and therapeutic challenge.

In the present study, we have shown type 2 MI is relatively common among patients hospitalized in general medical wards. Patients with type 2 MI were older and had similar comorbidities compared with patients with type 1 MI. These patients were less prescribed medical therapy and coronary intervention. They had higher 1-year mortality rates, but after accounting for age and sex differences, this association lacked statistical significance. Further studies assessing therapeutic strategies among patients with type 2 MI are required.

## Author contributions

**Conceptualization:** Chagai Grossman.

**Data curation:** Lee Gilad, Gil Neuman, Fadia Assad.

**Formal analysis:** Ariel Israel.

**Investigation:** Nadav Furie, Chagai Grossman.

**Methodology:** Nadav Furie.

**Resources:** Fadia Assad.

**Supervision:** Ilan Ben-Zvi.

**Validation:** Lee Gilad, Gil Neuman.

**Writing – original draft:** Chagai Grossman.

**Writing – review & editing:** Chagai Grossman.
